# The Hospital. Nursing Section

**Published:** 1907-02-09

**Authors:** 


### The Hospital.

Nursing Section.

Contributions for "The Hospital," should be addressed to the Editor, "The Hospital" Nursing Section, 28 & 29 Southampton Street, Strand, London, W C.

No. 1,065 —Vol. XLI . SATURDAY, FEBRUARY 9, 1907.

### Notes on News from the Nursing World.

### DISTRICT NURSES AND SPOTTED FEVER.

On another page will be found an account of the epidemic of spotted fever in Belfast, together with details of how it is nursed, and a concise description, by our correspondent, of the symptoms to be noted on the first appearance of the disease. The description is extremely valuable in view of the strong appeal of the Belfast Coroner at an inquest on Monday to parents to send for medical assistance the moment a child becomes ill. This, he stated, is the only means of stamping out this disease. We commend his remarks especially to district nurses, who, having read our contributor's article, will be in a position not to lose any time in notifying a case to the authorities, if they have even a suspicion that it is spotted fever.

### ONE NIGHT NURSE FOR 62 PATIENTS.

There has been correspondence between the Local Government Board and the Alcester Guardians in reference to statements made by a former assistant nurse. They were set forth in a letter sent to us by the nurse, under the heading of " An Incident in a Poor-law Infirmary," in our issue of January 19th. The Guardians rely upon the defence of the medical officer, who says that the principal definite allegations as to the patient who got out of bed are much exaggerated. He adds: " No doubt the nurse could have coaxed the patient back to bed had she taken the trouble." But " coaxing " is frequently a lengthy process, and while it was going on any of the other sixty-one patients, including seven mental cases, might be needing urgent attention. The medical officer gives away his case, however, by his intimation that he " considers one night nurse sufficient " for sixty-two patients.

### NURSING THE VICTIMS OF WILD BEASTS.

There are many features of interest in the article contributed to our columns this week on the nursing of injuries by wild beasts in British Central Africa. This is the kind of experience which cannot be gained in the United Kingdom, where the hippopotamus does not wander about, the crocodile is not likely to make a sudden appearance, nor the scorpion to inflict its poisonous sting. But if it could, a nurse would not be called upon to treat the patient suffering from an attack from either of these creatures; she would have the advantage of acting under the directions of a medical man. Our contributor, who has been stationed at Kota Kota, on the shores of Lake Nyassa, had to do the best she could in the absence of a doctor, who only visited the hospital " two or three times a year." So far as we can judge from her story, she did exceedingly well, and displayed an amount of resourcefulness which tends to show not only that she is a born nurse, but that she has been thoroughly well trained.

### SUICIDE OF A PROBATIONER.

An inquiry was held last week into the death of Margaret Cathleen Roberts, a probationer at Carshalton Cottage Hospital, who was found to have committed suicide by taking a large dose of strychnine. The evidence given included that of the sister of the deceased, a nurse at Croydon Hospital, who said that the latter was twenty-two years of age, was very passionate, and, when excited, would fly into a temper very easily. The matron of Carshalton Hospital stated that on the day the girl died she had to speak to her several times for various things, and at last sent her to her room. In the afternoon the probationer rang twice in five minutes, and when the matron went up to her she began screaming and declared that she had taken strychnine. The drugs were in a cupboard just outside her door. The matron administered an emetic and sent for the doctor. One of the jurymen suggested that the drugs ought to be kept under lock and key, but Dr. Cressy rejoined that it was essential that they should be accessible when wanted, and also that they were in the safest place that could have been selected. To show how determined the deceased was, he mentioned that she not only unlocked the cupboard containing the drugs, but after she had taken the poison, put the bottle back again. The coroner thought that the matron was bound to observe discipline, and did everything she possibly could in a trying crisis. It is essential to provide cupboards for poisons in hospitals, and to keep them locked.

### FRICTION AT A LIVERPOOL INFIRMARY.

The Highfield Infirmary Committee proposed to increase the salary of their doctor from £100 to £125 per annum. Opposition was made to the proposal at the last meeting of the Select Vestry because the nurses at Highfield had joined together to memorialise the Vestry complaining of the doctor. They stated that he did not assist them in maintaining discipline in the wards. One of the members of the Vestry explained that, according to the doctor's statement, all the trouble arose because the nurses wished to punish patients against the doctor's advice, and set themselves up as censors of the doctor's conduct. He went on to assert that " an egregious blunder had been made in the first instance by putting a matron instead of a doctor at the head of the Highfield Infirmary. He was afraid there would be nothing but friction so long as a woman instead of a medical man remained at the head of the institution." The whole of the circumstances may be investigated. The four nurses are ordered to appear at the next meeting of the Select Vestry.

### A DIRTY MIDWIFE IN CORNWALL.

A CASE which indicates that the provisions of the Midwives Act should be more strictly enforced in Cornwall has occurred at Truro, where the Coroner inquired into the death of the female twin child of a single woman, which died a few hours after birth. The mother was attended in her confinement by an old woman who was not registered as a midwife. The Coroner observed that he was sure no committee would grant the woman a certificate. Her hands were not fit to touch a person in confinement, and if she went before a committee in such a state she would not have the slightest chance of getting a certificate. He asked her to show the jury her hands. The woman declined to do so, and the Coroner thereupon added that he considered it very disrespectful to the court to come before them in that disgustingly dirty state. In returning a verdict that the child's death was due to premature birth, the jury added a rider that the attention of the Clerk of the County Council be called to the fact that there were women practising midwifery in Truro without certificates.

### THE MIDWIVES' ASSOCIATION.

THIS Association has been formed with an office at 9 Albert Square, Manchester, which is the office of the Women's Trades Union Council, "for the mutual benefit and protection of midwives." All registered midwives are eligible to become members. The entrance fee is one shilling and the weekly contribution is fourpence, in return for which financial benefits to the extent of six shillings and sixpence per week for the first four weeks, and five shillings a week for the second four weeks are offered "during cases of suspension," when not caused by the fault of the member. Members must pay contributions for 26 weeks before being entitled to the benefits, and they may, subject to the approval of the Association, obtain legal advice. There is no indication as to whether this Association is registered as a friendly society, or of the security provided for the payment of the weekly benefits. Not even the names of the bankers are given in the prospectus and leaflet which have been sent us. We are unable to ascertain how far this Association is a trade union, or whether this latter object is the main one for which it has been founded. If the Association is to be continued, it is essential that full information on these points should be forthcoming without delay.

### AN IMPORTANT APPOINTMENT.

THERE were no fewer than ninety-three applications for the post of superintendent of nurses in the education branch of the Public Health Department of the London County Council, and at the meeting of the Education Committee Miss Helen L. Pearse was elected at a commencing salary of £200 a year. The conditions of this new appointment are that it shall be held during the pleasure of the Council; that the superintendent nurse shall give her whole time to the duties of her office, and shall not be allowed to take any private business; that any fees received by her, either as a witness or in any other capacity, shall be paid to the Council; that she shall resign her appointment on marriage; that she shall enter into an agreement that she is appointed to and accepts the office upon the understanding that on retirement she shall not be entitled, and shall not make any claim, to any retiring allowance under the Superannuation Act, 1866; and that she shall submit to the Council's regulations in respect of the Superannuation and Provident Fund as now existing or as hereafter to be amended in pursuance of the Council's resolutions of October 16, 1906.

### GREAT NORTHERN CENTRAL HOSPITAL.

IN consequence of the appointment of Miss Helen L. Pearse to the post created by the London County Council, the office of matron of the Great Northern Central Hospital becomes vacant. Miss Pearse, who was only elected head of the training school at the institution in Holloway Road in July 1905, began her nursing career in 1889, when she was trained for a year at the Cambridge Nurses' Home. Having worked at the Home for two years as a private nurse, she left in order to obtain a full training at St. Bartholomew's Hospital. Here she remained for five years, when she was appointed superintendent of nursing at Lambeth Poor-law Infirmary. Two years later she quitted London to assume the post of superintendent of nurses at the North Stafford Infirmary and Eye Hospital, which she held for four years when she returned to London.

### TESTIMONIAL TO THE MATRON OF DARLINGTON HOSPITAL.

AT the annual meeting of the subscribers to the Darlington Hospital and Dispensary on Monday reference was made to the retirement of Miss Hunt, the matron. Warm tributes were paid by various speakers to her work, which was described as a labour of love; and it was decided unanimously to present a testimonial to her. A letter has been presented to Miss Hunt by Mrs. Edwin Pease and Mr. Edward Hutchinson, who were members of the Committee by which she was elected more than twenty years ago, stating:—"After above twenty years of devoted service to the hospital, and to the patients who have come under your charge during that period, the breaking of old ties must be very painful, and we all feel that the hospital is losing the superintendence of one who for so many years has given her best work to the cause of the suffering. The Committee recognise that while every care has been given by you to the patients, the comfort and training of the nursing staff and domestic servants has been efficiently promoted, and that by this, in conjunction with the able staff of medical men, our hospital has been raised to its present high position."

### OPENING OF A NURSES' HOME IN TORONTO.

ON Monday a new residence for the nurses of the Hospital for Sick Children at Toronto was opened. The building is the gift to the hospital of Mr. J. Ross Robertson, proprietor of the *Evening Telegram*, and the ceremony was attended by a very large number of prominent citizens. The lady superintendents of several New York training schools were present. Mr. Goldwin Smith delivered an address, and formally declared the building open. We hope to publish a plan of this building and to fully describe it.

### MILDMAY NURSES IN JAMAICA.

AT the time of the earthquake in Jamaica two of the four Mildmay deaconesses on the island were engaged in nursing work. They are known respectively as Sister Martha (Miss Stanley) and Sister Adelaide (Miss Wood), and are highly spoken of at the Mildmay Mission Hospital. Miss Wood was trained at St. George's Hospital, London, and worked for some years at the Mildmay Hospital as out-patient sister. Miss Wood has been in Jamaica two years, and Miss Stanley several years. Confidence is expressed that they have proved of the utmost service from their experience in tending the injured and suffering.

### THE FINANCIAL POSITION OF THE NURSES' CO-OPERATION.

THE Finance Report of the Nurses' Co-operation for 1906, which was adopted at the ordinary general meeting of members on Tuesday, shows a decrease of £120 11s. 2d. in the gross receipts from patients, and a decrease of £59 4s. 5d. in the sum paid to nurses in the year 1905. The income of the Society derived from the commission on fees amounted to £2,697 17s. 4d., or, together with interest on investments and deposit with bankers, £2,819 0s. 8d., as against £2,845 11s. 10d. in 1905. After payment of all working expenses there is left an excess of income over expenditure of £720 5s. 11d., as against £830 11s. 4d. in 1905. As in 1905, the sum of £500 has been invested, making the amount of the investments in the name of the Nurses' Co-operation close upon £3,500; while the cash balance last year was £1,340 5s. 1d., as against £954 12s. 6d. in 1905. In studying these figures it must be borne in mind that the Society reached a very high level of prosperity in 1905, when receipts from patients showed an increase of £747, and the sum paid to nurses an increase of £795 over the previous year. It is matter for congratulation that the large increase of business indicated by those figures has been maintained during the past year, subject merely to the slight fluctuations which are common to all undertakings of this nature.

### DISTRICT NURSES AND PATIENTS.

OWING to the kindness of various friends and subscribers, the matron and nurses of the Salford Royal Jubilee District Nurses' Home were enabled last week to give a tea party to a large number of their present and former patients. The Mayor of Salford granted the use of the Town Hall, and the Mayoress, who had entertained a party the previous evening, kindly allowed her decorations and plants to remain. Red art-muslin used as table-centres showed up the flowers and plants to advantage. At 5.30 cabs and bath chairs conveyed a few of the very old and helpless patients to the Town Hall, and by six o'clock the guests had all assembled. Tea on a liberal scale was then served, and was followed by a concert, which was thoroughly enjoyed, the entertainment being brought to a close by a magic-lantern exhibition. During the evening fruit was handed round, and when the guests prepared for home many of them received parcels of cakes and bunches of flowers for those who were not included in the invitation to the tea party.

### DEVELOPMENT OF THE SOCIAL UNION.

IN the annual report of the Nurses' Social Union some interesting details are given of its progress. Started in 1901, the Social Union held one meeting only in Somersetshire; in 1906 the number had risen to 19, and these were distributed in various centres in the counties of Gloucester and Somerset. The honorary members now include the matrons of Bristol General Hospital, Bristol Royal Infirmary, Taunton and Somerset Hospital, the Royal Mineral Hospital, and the Royal United Hospital at Bath, Yeovil Hospital, and the Free Hospital at Bridgwater. There is no doubt of the value of the Social Union, if only because of the opportunities it affords of bringing before an organised body of workers questions relating to the welfare of the people. The meetings held at different centres must have the desired effect of broadening the minds of nurses, and we can readily believe that if it were led and supported by medical men the Union might carry out the desire of its officials to wage a systematic war against such evils as infant mortality and tuberculosis.

### A SUBSCRIPTION DANCE AT LEEDS.

ON Tuesday last week at the Leeds Union Infirmary the nurses held their subscription dance in the dining-hall of the Imbecile Block. The entrance-hall and corridors were prettily decorated. There were about eighty present, and dancing commenced about eight o'clock, and was kept up until half-past two, the assistant doctors acting as masters of the ceremonies.

### ROYAL VICTORIA TRAINED NURSES ASSOCIATION

AN examination was held at Melbourne, Ballarat, and Bendigo by the Conjoint Board of Examiners of the Victoria Trained Nurses' Association on December 5 and 6. Sixty-eight candidates presented themselves, and fifty-nine were successful. How far the limited population of the colony will find work for professional nurses, with a yearly increase of from 100 to 120 members, remains to be proved. In the circumstances the suggestion that a nurse should cultivate some hobby between her cases, so that, if necessity arises, she could fall back upon it as a means of earning a living, may find favour.

### SHORT ITEMS.

THE number of cases attended in the past year by the staff of midwives attached to the Royal Maternity Charity, 21 Finsbury Square, was 2,651; but the number of infants born was 2,700, there being twins in 49 of the cases. Eighty of the infants were still-born, the maternal deaths were 8, and the infants 43.—A number of the Church Army Mission sisters are now learning training at Princess Christian's Maternity Home at Windsor. After they have passed the examination of the Central Midwives Board and received their certificates they will be known as "Princess Christian's Church Army Maternity Nurses."—An address on the Royal National Pension Fund for Nurses was given by the Secretary, Mr. Louis Dick, at the Nurses' Co-operation, 8 New Cavendish Street, on Friday last.

### The Nursing Outlook.

"From magnanimity, all fears above;
From nobler recompense, above applause,
Which owes to man's short outlook all its charm."

### POOR-LAW GUARDIANS AND DISTRICT NURSING.

It is considered probable, by those who have studied the question of poor-law relief as it exists to-day, that one result to be hoped for from the labours of the Poor-Law Commission, at present sitting, is the abolition of poor-law guardians. No doubt, in the past, boards of guardians have done a considerable amount of work, and for the most part they have done it fairly well. The tendency of the day in matters of local government is, however, to centralise as much as possible, with the object of securing better control, uniform practice and greater efficiency. If, for instance, the poor-law guardians were to be abolished, and the whole of their duties transferred to a committee of the county council, the present expense of administering the poor-law should be materially reduced, and the working of the poor-law system should tend more directly, to help the really deserving poor. Further the loafers and the idle, who at present gain advantages under the existing system which tend to foster this type of undesirables and to increase its numbers, would be treated with the necessary severity. If the county councils took over the work of the guardians, a great saving might be eff cted by closing unnecessary buildings, and the institution of one common system could not fail to improve the discipline, and to strengthen the administration, to the advantage of the ratepayers and of the poor themselves.

We have been led to these remarks by the difficulty, which is constantly recurring in the consideration of the position, which boards of guardians should take up, in regard to district nursing. By way of illustration we may take the case of the Chorley Board of Guardians. We gather that there are twenty-six townships under the jurisdiction of this Board, in only six of which are there district nurses working at the present time. In the Chorley township there is a district nursing association, to which Colonel Silvester has left a legacy to defray the cost of a district nurse, providing the expenses of a second nurse are forthcoming within twelve months of his death. Arising out of this circumstance application was made by the District Nursing Association to the Chorley Board of Guardians, asking the latter to subscribe from £10 to £15 a year towards the cost of the second nurse referred to. In the discussion which arose, objection was raised to any grants being made for district nurses, on the twofold ground, that, in addition to Chorley, five other townships had district nurses, and that if a grant was made to one all should have grants. Secondly, that there were twenty other districts where no nurses are employed, the ratepayers of which would have to contribute to these subscriptions and must so suffer an injustice. It is fair to assume, that where no district nurse has been engaged the ratepayers are either remiss in their duties, or belong to a class, or have other arrangements which render the services of such a nurse unnecessary to the residents. In either case, what injustice could arise from the guardians making a reasonable grant to the poorer or more efficient districts which employed nurses to attend upon the poor? As to the first objection, it is clear that if the guardians subscribe to one, they must subscribe to the support of all the district nurses in the area over which they exercise jurisdiction.

Now Guardians have authority to subscribe to public institutions and charities working within their area, which, by their work, tend to diminish the cost to the ratepayers of providing for the poor. We have pointed out, on more than one occasion, that a district nurse of the best type deserves well of all classes of the community. The advantages attaching to her work with the poor in all communities are great and ever extending. She speedily introduces neatness, order, method, sanitation and comfort into the humblest dwellings. In this way the district nurse is the handmaid of the medical officer of health and his staff, and she should become the handmaid of the boards of guardians in matters of relief in every district where the poor-law is intelligently administered. We believe that if district nurses were to be encouraged by boards of guardians, and their numbers increased, that the charge which such increase might entail upon the ratepayers through the subscriptions of the guardians, would be relatively unimportant compared with the value of the services which each district nurse might render to the poor-law in matters of relief, and to the health authority in many directions. If the work of the boards of guardians was to be relegated to a committee of the county council, we make no doubt that this important fact would be speedily recognised, and that every community would secure the services of an efficient district nurse. In an old system such as ours, prejudice and custom cause impediments to progress which new communities cannot appreciate. The shortest cut out of the difficulty is reorganisation, and the county council in this matter is, in our view, the best solution of the difficulty. In any case the more intelligent the ratepayers, and the more closely they examine into this question, the more certain is it, that, a large majority must become favourable to the grant of money by the guardians, or any substitued authority, to the support of an adequate system of district nursing.

### Nursing in Tropical Climates.

By ANDREW DUNCAN, M.D., B.S., M.R.C.P., F.R.C.S., Fellow King's College, Lecturer on Tropical Medicine at the London School of Tropical Medicine, and the Westminster Hospital.

### VI. ENTERIC FEVER.

(*Continued from page* 250.)

*Woodbridge Treatment of Enteric Fever.*—This treatment is recommended by Dr. Woodbridge, of America, who quotes a case-mortality of only 1.90 per cent. amongst 7,857 cases of typhoid fever, and an average duration of illness of only 12.7 days has been tried in India. Briefly, the treatment is "a general or intestinal antiseptic and eliminant one, and consists in giving very frequently (every fifteen minutes during the wakeful portion of first forty-eight hours) small doses of varying formulæ or podophyllin resin, and calomel, combined with such antiseptics as guaiacol carbonate, menthol, eucalpytol, and thymol, the indications being to produce free evacuation as early as possible, and by subsequent varying doses to keep the bowels regular. The maximum number of doses in the twenty-four hours, supposing the patient not to sleep at all, would be ninety-six."

Major Hendley, I.M.S., who has tried this method, states that he never succeeded in getting down more than seventy doses in twenty-four hours. His experience was favourable; but one does not know which to pity the most in this method, the unfortunate patient who has to take his medicine every quarter of an hour, or the unfortunate nurse who has to administer it.

We will now consider the methods of preventing the spread of enteric fever that should be carried out by the nurse, both as regards infection generally and infection of herself.

*A. Measures to Prevent Infection Generally.*—The enteric bacillus gains an entrance into the body through the digestive canal, being swallowed with food or drink, and occasionally through the lungs. Once swallowed, it multiplies in the bowel, and in so doing causes the changes in the intestines of which mention has been made, and then leaves the body again by the stools and urine. When it has entered by the lungs the bacillus will be present in the sputum coughed up; when it leaves the body by the excreta it may infect the bed-clothes, the utensils receiving the discharges, the hands of patients and of the nurses, unless kept clean and disinfected. It will also, of course, infect any food and drink with which it is brought into contact.

To take the evacuations first. As regards these an antiseptic should be placed in the bed-pan before use. This may consist of a carbolic acid 10 per cent. solution. After the excreta has been passed add some more of the disinfectant, so that the quantity may equal the bulk of the excretion. Thoroughly stir it up, then cover over with a cloth, and let the antiseptic act for at least half an hour before the stool is buried. Other disinfectants that can be used are a $\frac{1}{10}$ lysol solution or a 1-500 corrosive sublimate. This by many is not advised, as it hardens the albuminous material outside the fæcal masses, but personally I have never found any failure from this reagent. The nurse should bear in mind the necessity of disinfecting the urine. The sputum should be disinfected in the same way. After being thus treated, the discharges are to be buried in the earth; the depth at which they are to be buried is variously recommended. Some authorities say at least two feet, whereas the late Dr. Vivian Poore was an advocate for shallow burial; and, in the only instance falling under my observation of this way of disposal of the fæces, the results were excellent. The best method, however, of treating the excreta is to burn them, mixing them with sawdust. Should the discharges containing the bacillus be thrown on the ground without being disinfected, through the carelessness of the "sweeper," the well in the compound would be infected should the germ reach it. The dangerous consequences ensuing on such a course of action were well shown by the extensive epidemic that occurred in the town of Plymouth in Pennsylvania in 1885. Here a man convalescent from enteric fever was found by the Committee who were appointed to investigate the outbreak to be living in the only house on the banks of a mountain stream of great purity that formed part of the water-supply of the town. Only the people supplied from this source suffered. During the illness of this man his stools were thrown on the snow on the ground within a few feet of the above stream. This was in February and March. At the end of March the snow began to melt, and in April this melting was completed by the occurrence of frequent showers with mild warm weather. The first case of enteric was reported on April 9. Within the next five days thirteen more cases occurred, and then a large increase took place, a great outburst flaring up about the middle of the month. More than 1,000 cases occurred in all.

The stools should be disinfected as soon as they are passed, as their infective properties appear to be developed in about twelve hours.

All utensils, feeding-cups, enema-syringes, etc., used by the patient must be scrupulously disinfected. The soiled bed-linen and dress of the patient must be likewise so treated, and they should be also disinfected at once, lest any infectious particle dry on them, and subsequently be wafted off into the air as dust, and then settle on some food and infect it. The linen can be put into a large tub and soaked in disinfectant solution, afterwards boiled, and then dried in the sun. Burn all small pieces of linen that may have been in contact with any discharge.

Lastly, we have before alluded to the part played by flies in propagating the disease. They should be kept excluded from the chamber of the sick by attention to the "chicks"; and the stools, during the time they are kept exposed to the action of the disinfectant, before they are disposed of, must be carefully covered over with a cloth.

[As regards the infection that may be carried by the patient himself for some length of time during convalescence by the urine, urotropine in 7-grain doses should be administered three times daily for at least a month.]

We now come to the precautions to be undertaken by the nurse herself. A golden rule in the East is never to drink any unfiltered water. The only efficient filters are the Berkefeld and the Pasteur. The Berkefeld Company sell an excellent portable one.

Another method of disinfecting the drinking water is that of Parker and Rideal, who state that the bacillus typhosus is killed after five minutes' contact with bisulphate of soda in the proportion of 15.5 grains to the pint of water. Fifteen minutes' contact is more advantageous. Firth, however, says that forty-five minutes' contact is necessary in order absolutely to sterilise the water.

The nurse should not wear her nails long, and after attending to the patient in any way, she should always wash her hands well with a brush, and then immerse them in some disinfectant solution for at least five minutes, and then rinse them in clean water. She should always wash her hands and disinfect them before taking her own meals, which must not be eaten in the sick chamber.

*Antityphoid Inoculation.*—Professor Sir A. Wright has introduced this method of prevention. What is the evidence in its favour? It would appear to me to be overwhelming. The published statistics from six hospitals, for instance, in the South African War—namely, those of the Ladysmith, Princess Christian, Portland, Scottish National Red Cross, Kroonstadt, and Harrismith Hospitals—all concur in showing a much less incidence on the inoculated than on the uninoculated. But an example of the benefit thereby accruing, which is more apposite as far as nursing is concerned, was shown by the epidemic in Maidstone. Here, of 120 nurses who were not inoculated, 16 were seized with enteric, whereas of 84 nurses and atendants inoculated, not one was attacked. As regards the advisability of being inoculated as a preventive against enteric fever, I can only state that if I were again about to embark on a career in India at the age that one enters Indian service, I would most certainly be inoculated myself.

Summary of the Especial Points in Nursing a Case of Enteric Fever.

1. Do not pile on bed-clothes. A sheet is quite sufficient protection from cold.
2. From the beginning of the case take precautions against bed-sores.
3. Keep the patient's teeth, nose, and mouth clean with a disinfectant.
4. Remove all soiled bed-linen at once, and disinfect them.
5. Disinfect the fæces and the urine directly they are passed. Let them be exposed to the action of the disinfectant at least half an hour before disposal.
6. Alter the position of the patient in the bed from time to time, so that too much pressure be not exerted on one part of his body.
7. It is not necessary to wake the patient up always for his food if he is sleeping. If he is in a state of stupor his food must be given him.
8. If there be signs of hæmorrhage in the intestine before the doctor comes
   - (*a*) Stop all food;
   - (*b*) Keep the patient absolutely at rest;
   - (*c*) Apply an ice-bag to the right iliac region.
9. Report at once—
   - (*a*) If any curds found in the stools;
   - (*b*) If any pain in the abdomen;
   - (*c*) If any sudden rise or fall of temperature.
   - (*d*) If any hæmorrhage.
10. Always wash your hands with some disinfectant after attending to the patient, and before taking your own meals.
11. Do not take your meals in the sick-room.
12. Keep all flies away from the patient.
13. Never yield to the patient's hunger and give him more than he is ordered.
14. The patient should never be left alone.

### The Nurses' Clinic.

### ERYSIPELAS.

Erysipelas—or St. Anthony's Fire, as it is sometimes called—does not occur now so frequently in England as it did before the days of antiseptics and asepsis; but even in present times, when all precautions are taken, it appears sometimes.

Generally it starts from a wound, scratch, or skin abrasion of some sort, though this is not always the case, as some people are subject to it without any wound at all, and it is then called medical or idiopathic erysipelas, in contradistinction to surgical erysipelas, or that arising from a wound.

This complaint is distinctly contagious, and in nursing it the nurse must be most careful not to have an open sore, cut, scratch, or even pin-prick on her hands, or she will run great risk of taking it.

Erysipelas is a peculiar form of inflammation of the skin. If not determined by a wound, it most commonly attacks the face, starting by one or more red spots, which spread rapidly, making the whole surface affected bright red, very much swollen and œdematous, with one or more blebs filled with yellowish fluid.

The eyelids and ears are very puffy, resembling bladders, and from the eyes runs a watery discharge. It is a painful disease, and often the glands of the affected parts are swollen and tender.

If a rash starts at a wound, the greater precaution must be taken by the nurse when doing the dressing. All soiled dressings and discharges must be burnt at once, and receivers, forceps, probes, etc., that are being used for such a case should be kept quite separate, even when sterilised.

The nurse, of course, must be most careful about washing and disinfecting her hands after touching a case.

If the disease has attacked the face, especial attention must be paid to keeping all cups, spoons, etc., for that patient only.

Different doctors have their different methods of treat-

THE NURSES' CLINIC.—*Continued.*

ment, but the most popular appears to be dressing the wound and then powdering the red inflamed part with starch, zinc, or boric powder, and keeping the infected area covered with a mask of cotton-wool if on the face, or pads of cotton-wool lightly bandaged on any other part. The patient's strength must be kept up with plenty of milk, beef-tea, chicken broth, and sometimes port wine or brandy may be ordered. Many medical men order tincture of iron, which seems by some to be considered a specific for erysipelas. The patient will probably be very weak, as generally a high temperature is run, frequently rising to 105°. After the fourth day, in a normal case, the temperature will probably drop.

Be most careful to keep the patient from any chills, as such invalids are very susceptible to pleurisy, pneumonia, and kidney trouble.

The patient should be warmly covered up, kept in an even temperature, without draughts, and all symptoms or changes must be carefully noted and reported to the doctor, such as pains in the heart, or cough, etc. In some cases, especially of facial erysipelas, they get, occasionally, a "murmur" in the heart during the early days of the disease, which passes off later on.

For idiopathic erysipelas lead lotion is frequently ordered to be applied to the face in the shape of a lint mask dipped into the solution and applied to the face. This seems to give great relief to the pain and swelling, which subsides very soon after its application. I had several bad cases of medical erysipelas abroad as a complication of typhoid fever. One case was very remarkable. The patient had had typhoid fever with two relapses, and after two and a half months was fairly convalescent, being already on light food, when one morning at 10 A.M. he told me he had been stung by a sandfly. I looked at the right side of the chest and saw it was red and slightly swollen, and had the appearance of having been stung, but after careful examination I could not find the smallest puncture of a sting. Lead lotion compresses were applied, but the patient still seemed very uneasy, and two hours later the swelling and redness had spread to the left side of the chest and the swelling had increased greatly. The temperature was now 105°, and the patient was wandering in his speech. From this time on he got gradually worse, and died at 6 P.M.

Another case was also a typhoid convalescent, who started erysipelas down one side of the face. He got a large sloughing abscess in the neck, and was in hospital with it over two and a half months, but recovered eventually. In both these cases the cause of the erysipelas could not be traced, as there had been no other cases in the ward at the time.

In medical erysipelas the native patients resort greatly to "charms" for curing themselves. An old woman comes around with a bit of red twill and a blest candle, and, going through a ceremony with many incantations, is supposed to have charmed away the disease; and, strangely enough, I have seen some very bad cases completely cured in twenty-four hours after this performance.

Medical erysipelas is seldom fatal, and, if properly nursed, generally gets well quickly; and though a run of such cases means continual work, such as changing dressings, fixing masks, applying lotions, and seeing that nourishment is taken properly, yet they are very satisfactory, as they yield to treatment very quickly. Some people get the complaint again and again at short intervals, in the same way as others suffer from hay-fever. After a time one gets very used to these cases, and it is most necessary for a nurse not to get slack over the disinfecting part of the nursing, or she may learn to her own cost what carelessness in this respect may lead to.

The surgical erysipelas fatal cases are not so rare, but generally occur in old people, especially if they are suffering from any virulent complaint or from chronic alcoholism. When the red rash dies off, or rather turns brown, the blebs dry up and peel off as in scarlet fever; and it is wise to paint the face with oil or simple ointment, to keep the particles from flying about.

If the scalp has been affected, probably all the hair will fall out.

Sanitary surroundings and cleanliness of person are to be specially recommended to patients who are subject to a frequent recurrence of the complaint, as dirt is often a great factor in starting it.

### Incidents in a Nurse's Life.

### A PROVIDENTIAL LAPSE OF MEMORY.

It is seldom indeed that any lapse of memory has beneficial results in hospital life, and within my recollection only one example has occurred. I was in one of the large London hospitals on night duty when, early in the evening, an accident case was admitted. A man of middle age had slipped between the platform and a moving train, and the flesh had been cleanly cut away from the lower leg, leaving the tibia exposed for many inches. He was returning from the races, where he had been successful in making a big pile, which, however, had all gone when I turned out his pockets, except a few shillings. He had been drinking heavily, but the shock had sobered him, and he demanded to know how much was left out of the £30 he had taken, and wondered vaguely where the rest had gone.

Once in bed, he was put under an anæsthetic and his wound scrubbed with soap and water and strong disinfectants; but the dirt and train oil were ground deeply into the flesh, and in two days it was streaming with pus.

On the third night upon coming on duty I was amazed to see him in a theatre-gown, with his head shaved and compressed, and to learn that he had developed tetanus and was to be trepanned at once, that the antitoxic serum might be injected into the brain. I was unable to be present in the theatre, no staff nurse being permitted to leave her ward at night; but the dark hours were anxious ones, and the terrible spasms took hold of him again before morning. He was put on the danger list, and a woman and child came up and stayed with him almost continuously.

On the third night after the operation he was very weak and partly unconscious, and it did not need a greatly experienced eye to see he would not last till morning. The young woman and the child remained at the bedside for a while and then departed. He showed some signs of consciousness when I spoke to him or tended him in any way, but he was terribly feeble, and before daylight came the faint spark had gone out and life's debt was paid. Sister, coming on duty in the morning, stopped at the empty bed.

"Nurse, did you notice the folded paper in the rack over the bed?" she said quietly, while I saw her face change.

"Yes, it's with the notes of the case and the chart, sister; I supposed it was part of the history."

"Unfortunately it was a will a lawyer brought in yesterday. The patient was not conscious at the time, and he begged me to get it signed if any way possible before he died. I'm afraid it was my fault it was forgotten."

I could see she was more vexed than she cared to allow at her lapse of memory, and I said reassuringly :—

"I don't think there was any moment of the night when his brain was sufficiently clear or his hand steady enough for him to have put his signature to anything."

Later in the day we learned the contents of that will. Had he by a great and dying effort signed it his wife and family would have been left penniless, and the woman and child who had no legal claim would have come into a small fortune.

### Nursing in British Central Africa.

### INJURIES BY WILD BEASTS.

During the last few years I have had to treat several cases of injury by wild beasts. I have been stationed at Kota Kota, on Lake Nyassa. Our doctor visits us two or three times a year, and in his absence I have to do the best I can.

### A Hippopotamus Bite.

I was told one Saturday that a man had been bitten by a hippopotamus at a place about twenty-five miles distant. The only means of getting there was by being carried in a hammock, and it was a whole day's journey distant. My informant was not at all sure that I should find the injured man at the village at which the accident occurred, as his relatives would probably have conveyed him up to the hills if he were still living. It was not worth while going so far for anything so uncertain, so I sent a message to say that I would admit him into hospital if he cared to come. On the following Tuesday morning while I was busy in the dispensary a tall native walked in and stood silently awaiting his turn. I glanced up at him and perceived that he was a stranger and was looking exceedingly ill, so I told him to sit down and asked him what ailed him. For answer he raised a dirty piece of calico that was draped like a shawl over his right shoulder and disclosed the fact that his arm was missing. The hippopotamus had bitten it clean off about four inches above the elbow. The man had gone into the river to fetch his fish-trap and found a large hippo. in the act of destroying it. He had attacked the beast with his spear, but it turned quickly, and seizing him by the arm swam off. Its teeth met through the arm, and the man dropped, and with a great effort swam to the shore. His friends had fixed on a tourniquet made of bark-rope, tightened up with a stick; they had applied a kind of bird-lime to the wound, and recommended him to come to me. It had taken him about three days to perform a journey which he could have ordinarily accomplished in one.

### Treatment of the Wound.

The wound, or the bird-lime, or both, had proved very attractive to flies, and the first three days after his arrival were spent in trying to cleanse the wound from maggots, which developed in three distinct crops. The native dressing was excessively sticky and adherent, and in spite of continual fomenting and soaking I was unable to get it off for several days. I was afraid to use any force in removing it lest I should cause any of the severed arteries to bleed. The wound was well packed with sulphur and cyanide gauze and the patient's strength kept up with nourishing diet and stimulants. The wound cleaned up wonderfully, and as soon as it was quite clean it began to heal rapidly. In three months it was soundly healed, and the patient returned home.

### Injured by a Crocodile.

I was asked to come and see a man who had been mauled by a crocodile. He had been sleeping outside his hut, and the crocodile had seized him by the hands. He shouted out, and his wife ran out of the hut and drove the creature off with a hoe. There were several wounds on both hands, the top of one thumb was nearly severed, and the right forearm was fractured, both radius and ulna. The patient was admitted into hospital, the wounds well cleansed, and, where possible, stitched up—there were seventeen stiches in all—and the fracture reduced as well as possible under the circumstances. To my astonishment the patient did well. The only wound that suppurated was that in the thumb, the tip of it sloughed off, but the stump healed all right.

### Poisoned by a Scorpion.

The following case of scorpion sting is interesting, though perhaps it can hardly be called an injury by a "wild beast." A prisoner working in the garden felt suddenly a sharp pain in the knuckle of his middle finger, and found he had been stung by a scorpion. His finger was very painful, and he complained of it to the warder when being locked up for the night, but no notice was taken of his complaint. Early in the morning, however, the warder was aroused by his cries, and found him in great pain. His whole hand and arm were terribly swollen, the skin tight and shiny, and looking as though it must burst. He was at once brought down to me. The exact spot where the poison had entered was not discernible, and the patient was wholly unable to locate it; could not even remember in which finger it had been, or I should have made a cruciform incision and treated it with pot. permang., as for snake bite. The only thing I could think of was to apply hot glycerine and belladonna fomentations at frequent intervals, and give the patient stimulants. These relieved the pain, but for twenty-four hours the swelling increased until the whole of the left side of his chest and back were greatly swollen. The pulse was less rapid, however, and the temperature remained as it was when the patient was admitted. Thirty-six hours after admission the swelling had very greatly decreased, and twelve hours later only the hand was swollen. The seat of injury was then visible, and suppuration was going on all round it. An incision was made, and carbolic fomentations applied to the finger. The patient insisted on going out before it was absolutely healed. A few months later he returned with an abscess on that finger reaching down to the bone. It was suggested that he should have an anæsthetic and have it scraped, but he absolutely refused. When seen a few months later the two first phalanges of the finger had sloughed right off, but the stump had healed.

### To Nurses.

We invite contributions from any of our readers, and shall be glad to pay for "Notes on News from the Nursing World," "Incidents in a Nurse's Life," or for articles describing nursing experiences at home or abroad dealing with any nursing question from an original point of view, according to length. The minimum payment is 5s. Contributions on topical subjects are specially welcome. Notices of appointments, letters, entertainments, presentations, and deaths are not paid for, but we are always glad to receive them. All rejected manuscripts are returned in due course, and all payments for manuscripts used are made as early as possible after the beginning of each quarter.

### Nursing Spotted Fever in Belfast.

FROM A CORRESPONDENT.

During the past few weeks much anxiety and alarm has been caused amongst the people of Belfast through the outbreak of "Spotted Fever," otherwise called epidemic cerebro-spinal meningitis.

Each day of late has brought reports of one or more fresh cases of the fever.

The cause of the outbreak is as yet unknown, and the sanitary authorities have not so far been able to get at the root of the trouble. The epidemic has not confined itself to any particular locality, nor have the cases been in the slums of the city. Most of them have occurred at distances from one another, and a good many of them in open and respectable streets. In one or two instances only have several children of one family been attacked.

The fever is said to be contagious in some way, but in what way is not clearly known, any more than the period of incubation from the time the disease germ enters the system until the symptoms show themselves. Up to the present time in most cases the onset has been apparently sudden. A few words may be said as to the symptoms which have been noticed in most of the cases since the outbreak of this epidemic. The patients in the first instance all suffered from severe headache and vomiting; the headache gradually got worse; after a few hours painful contractions of the muscles of the neck began, the pupils were widely dilated, there was flexion of the knee-joints, and rigidity of the muscles.

The second day small hæmorrhagic spots appeared on the legs and lower parts of the abdomen; in some cases there was violent delirium, the temperature in bad cases reached 106° F., the patients as a rule were highly sensitive to touch, every movement giving the most intense pain. After the first twenty-four hours, or even earlier in bad cases, the patient sank into a comatose condition, became cyanosed, with stertorous breathing, and died from thirty-six to seventy-two hours from the onset of the disease.

In the present epidemic the disease seems to be chiefly confined to children and young adults. The first case brought under notice was that of a young medical student, about twenty-three years of age, who lodged in a good locality of the city, was a student of the Queen's College, and attended classes at the Royal Victoria Hospital. He had been at the hospital as usual on the Saturday previous to his illness. On Sunday morning he went for a walk, feeling in his usual health except for slight headache, and said to one of his friends that he felt he was taking a cold. On Monday the headache was worse, and feeling sick and out of sorts, he was unable to leave his bed; during the evening a doctor was called, who found him in a serious condition. He was sent to the Royal Victoria Hospital without delay, and, in spite of everything that could be done, he died on the afternoon of Tuesday, having some hours previous to death sunk into a comatose condition, with stertorous breathing. As the disease had been diagnosed as "Spotted Fever," precautions were taken to disinfect the private ward in which he was nursed.

Another case a few days later was that of a young man of sixteen, a shop assistant in the city. He was admitted to the isolation wards of the same hospital, but he also died about forty-eight hours after admission.

Since then four children of one family contracted the fever, no symptoms being visible until within a few hours of death. The children went to bed at night apparently in their usual health; during the night two of them, who were sleeping in the same bed, became ill. One child was dead before a medical man could be summoned, and another was dying when the doctor came. Two other children of the family in the same house were next found to have acute symptoms, and later on in the day both died; a fifth child, complaining of headache, was at once taken to the Belfast Infectious Diseases Hospital at Purdysburn, where he was placed under observation, and is now convalescing from a slight attack of the fever. Since then a sixth child of the same family has developed the disease; he has also been taken to Purdysburn, as have all other cases since reported. Up to the time of writing the cases number 45, the number of deaths being 21.

With regard to the nursing of these cases. In most instances the head is shaved and the ice cap applied in the usual way; as the patient is generally collapsed the heat of the body is kept up by hot-water bottles and blankets. In some cases blistering the spine may be ordered, and lumbar puncture is often done by the doctor to relieve tension; in both instances the skin, etc., will require the ordinary aseptic preparation. The patient's thirst is generally very great, and as much fluid nourishment as can be taken may be given, as emaciation and exhaustion are rapid. When the muscles of the neck become rigid the patient is unable to swallow, and nasal feeding is the only hope of giving nourishment; stimulant also which will most likely be ordered may be given in this way.

The nurse requires to handle the patient with the greatest gentleness, as when conscious he is acutely sensitive to pain.

The patient as a rule loses control of the bladder and rectum, and the bed will require frequent changing; some patients suffer from diarrhœa, for which little can be done; others from constipation, for which calomel is often prescribed.

When the delirium is violent the physician sometimes orders morphine hypodermically, or chloral, which is given in the nasal feed or by rectum.

The nurse may also be required to give inunctions in the groin and axilla alternately twice daily of a mercurial ointment prescribed as an antiseptic.

The temperature, pulse and respiration must be charted four hourly.

When the case is favourable the temperature generally comes down in about a week's time after the acute stage, but is apt to go up again in a day or two. The convalescence is very slow and is attended by frequent relapses for the first six weeks, and it is said that those who recover sometimes suffer from a form of paralysis. The period of convalescence is said to occupy from two to six months, and tonic treatment is ordered. It is hoped that Belfast will soon be rid of this epidemic, which has already caused so much anxiety and distress.

### Where to Go.

The Dowdeswell Galleries.—At the Dowdeswell Galleries Mr. Oliver Hall is showing some charming examples of his recent work. Mr. Hall has some of the restful quality which gives the work of Constable so much charm, and his treatment of trees often suggests the work of that master. He is particularly happy in dealing with Yorkshire scenes, and the pictures of Knaresborough, Bolton Priory, and Fountains Abbey will bring pleasant memories to those who are familiar with the places. And to all the tender fidelity of the work will be welcome, as a change from the vagaries of the impressionist school.

### Meeting of the Central Midwives Board.

A Meeting of the Central Midwives Board was held at Caxton House on Thursday last week. Dr. Champneys was in the chair, and there were also present Miss Paget, Miss Wilson, Mr. Parker Young, Dr. Dakin, Mrs. Latter, and Mr. Ward Cousins.

The minutes of the last general meeting on November 29 having been passed, there were the reports of two meetings of the Standing Committee to be dealt with. At the first of these, on December 13, on further consideration of a letter, referred to the committee by the Board, from Dr. Vacher, county medical officer, Cheshire, enclosing a circular and copy of an advertisement presumed to be issued by a certified midwife, and asking the Board to deal with the matter, the committee recommended that the local supervising authority be requested to state whether in their opinion a *prima facie* case of malpractice or misconduct has been established within the meaning of Section 8 (2) of the Midwives Act. This was carried.

### Extension of the Act to Ireland.

A letter was read from the Assistant Under-Secretary for Ireland explaining that, under the present circumstances, when a great many nursing posts were open only to candidates who had obtained the certificate of the Central Midwives Board, nurses who had passed through the maternity training schools in Ireland were disqualified for such posts unless they went to the expense of coming over to England to obtain the Central Midwives Board certificate. He was, therefore, instructed to inquire, on behalf of the Lord Lieutenant whether there would be any objection on the part of the Board to steps being taken to amend the Midwives Act, 1902, so as to enable the Board to hold examinations in Ireland. This once more roused a discussion on the negative attitude adopted by Ireland in the first instance towards the Act and the inexpediency of sending examiners over to Ireland to certify midwives over whom the Board would have no further control. Finally, on a motion proposed by Miss Wilson, seconded by Miss Paget, it was decided to reply "that the Board recognises the difficulties of an extension of the Midwives Act to Ireland, but would look with favour on a new Act for Ireland framed on the lines of the present Act."

### The Amendment of the Rules.

In considering the amendment of the rules, many of which had been already dealt with and adopted by the Board, the committee recommended "that the Privy Council be asked whether the Board has power under the Midwives Act to frame a rule providing for the removal from the roll of the name of a woman wishing to retire." This arose from the fact that the Board has received a number of applications from *bona fide* midwives who do not feel competent to carry out the rules of the Act, but wished to retire with honour. At present the only means of removing their name from the roll is to convict them of malpractice or misconduct. The recommendation was carried, the chairman remarking that it was a very laudable desire on the part of the *bona fide* midwives; but Mr. Ward Cousins was suspicious that after their names were removed they might continue to practise in secret.

For the purpose of consideration of the method of revision of the lists of approved training schools, teachers, and midwives, and reporting thereon, it was resolved that a sub-committee be appointed, to consist of Dr. Dakin, Miss Paget, and Miss Wilson; and a meeting of this subcommittee was arranged for Thursday, March 7, at 2.15 p.m.

### The Notification of Still-birth.

The report of the Standing Committee, which met on January 24, called to the notice of the Board a letter from Sir Shirley Murphy, County Medical Officer for London, suggesting an alteration in the form of a notification of a still-birth, and it was agreed that the matter be considered on the final revision of the Rules.

### A Copenhagen Candidate.

A question arose as to the admission to the February examination of a candidate whose certificate had been signed by Professor Meyer, of Copenhagen. Although Professor Meyer is not a recognised teacher under the Act, his name, as the Chairman pointed out, would be a welcome and honoured acquisition to the list. The candidate who now wishes to obtain the certificate of the Board has had a longer and more thorough training than any we give to midwives in this country, and it seemed only just and right that the Board should be allowed to modify its own rules rather than refuse such an eligible candidate. A motion to this effect was put by Miss Paget, seconded by Mrs. Latter, and carried.

### The Penal Cases.

Certain charges were alleged against Mary James, Mary Lixton, Hannah Rhodes, and Sarah Scott by their respective local supervising authorities, but the Board decided that they should not be cited until the matter had been investigated more fully. Mr Parker Young and Mr. Ward Cousins were, however, of the opinion that the Board should do its utmost to remove from the roll such midwives as Mary James, who could neither read nor write, for, though her bag was found to be satisfactory and to contain the prescribed appliances, they were no good to her as she did not know how to use them. They maintained that it was a grave mistake for the Act ever to have admitted these women. It was further agreed that Elizabeth Webb and Lilian S. Williams be called upon to furnish an explanation of the charges alleged against them and that no action be taken in the cases of Sarah Elizabeth Ellison and Dinah Ann Peace.

The financial statement was not quite so satisfactory as it has hitherto been, and the Secretary informed the Board that it would soon be necessary to sell out some of their stock.

### The Case of Whitechapel Infirmary.

A letter was read from Dr. Larder, medical superintendent of the Whitechapel Union Infirmary, renewing the application of the Guardians for the approval of the infirmary as a training school. Owing, however, to the absence of Mr. Fordham, the motions relating to this question on the agenda were postponed. Some discussion arose as to the right of the Board to refuse their approval of any place as a training school on structural grounds, and the Chairman explained that the general feeling was that so long as the training given by a school was efficient, they had no right to refuse approval, as they had already "approved" some institutions which had no "structure" at all, but did their work entirely amongst out-patients.

The next meeting of the Board was fixed for February 28, at 2.45 p.m.

### Queen Victoria's Jubilee Institute for Nurses.

Miss M. Wilkinson has been appointed to Barford, Warwickshire; Miss Helen M. Weale, to Grimsby; Miss M. W. Leonard, to Rochdale; Miss G. E. Gomm, to Blaisdon, Gloucestershire; and Miss Lucy Davies, to Llangefni, Anglesey. Miss F. E. Whitfield has been temporarily appointed to Norwich as superintendent; Miss Madeline Naylor, to Harpurhey Home, Manchester; Miss S. W. J. Hadden, to Edensor; Miss Florence Green, to Gloucester; and Miss K. Berkeley Smith, to Hanley. Miss T. J. Lesser has been transferred from Hackney to South Tottenham, and Miss Dorothy Bracewell from Cheadle Hulme to Bolton.

### Everybody's Opinion.

[Correspondence on all subjects is invited, but we cannot in any way be responsible for the opinions expressed by our correspondents. No communication can be entertained if the name and address of the correspondent are not given as a guarantee of good faith, but not necessarily for publication. All correspondents should write on one side of the paper only.]

### A PROBATIONER LOSES HER SIGHT.

"J. B. F." writes from 43 Montpellier Terrace, Cheltenham: I beg to enclose a cheque for £1 for the nurse whose sight was so injured by a typhoid patient spitting in her face. I trust that she may make a better recovery than is anticipated, and be able to resume her career of usefulness.

The Secretary of the Royal National Pension Fund for Nurses writes: I enclose a postal-order for 2s. 6d. which I have received from Nurse R. Webb of the Camberwell Infirmary, Constance Road, East Dulwich, for the West Ham Infirmary probationer, whose case has been described in your columns.

### A MATRON'S APPOINTMENT.

The Matron of the City Fever Hospital, Grafton Street, Liverpool, writes that Miss O'Brien, the new matron of the Manchester and District Joint Isolation Hospital, was charge nurse and not assistant matron of the Grafton Street Hospital.

### BRITISH LYING-IN HOSPITAL.

Mrs. Gerald Maude, Hon. Secretary of the British Lying-in Hospital, writes: I shall be much obliged if you will allow me the use of your columns to state that a bazaar in aid of this hospital (which is greatly in need of funds) is arranged to take place at Prince's Skating Rink on May 29 and 30 next, under the special patronage of H.R.H. the Princess of Wales, H.R.H. the Princess Royal, and H.R.H. Princess Louise (Duchess of Argyll), and that the latter has graciously consented to perform the opening ceremony. Amongst the stalls will be one conducted by the matron and the nurses, at which all sorts of garments suitable for poor women and children will be sold, and, as there are many nurses throughout the country who have been trained at the hospital, I wish to say that, if they would individually contribute one or even two articles towards the stocking of the stall, such gifts would be much appreciated. They should be sent to me at 50 Onslow Gardens, London, S.W., not later than April 31.

### Appointments.

Bolton Borough Fever Hospital.—Miss Irene Webb has been appointed matron. She was trained at the London Hospital, Whitechapel, E., and has since been night superintendent at Grove Fever Hospital, Tooting, S.W., assistant matron at the Fountain Fever Hospital, Tooting, S.W., matron of the Fever Hospital, Leicester, and matron of the Fever Hospital, Gravesend.

Broadstone Jubilee Hospital, Port Glasgow.—Miss Minnie Templeton has been appointed matron. She was trained at the Glasgow Western Infirmary, and has since been sister at Greenock Infirmary.

Cancer Hospital, Fulham Road, London.—Miss Bridgett M. Powell has been appointed staff nurse. She was trained at the Royal South Hants Hospital, Southampton.

Hammermsith Infirmary, Wormwood Scrubs.—Miss Marie White and Miss Dora Williams have been appointed staff nurses in the maternity ward. Miss White was trained at the London Hospital, Whitechapel, E., and has been on the private staff. Miss Williams was trained at Bradford Union Infirmary, and has since done private nursing at Harrogate.

Melksham Cottage Hospital.—Miss Maud Constance Saunder has been appointed matron. She was trained at the Royal Albert Edward Infirmary, Wigan, where she subsequently held the posts of ward sister and night sister. She has since been assistant matron at Her Majesty's Hospital, Stepney, London.

Royal Chest Hospital, City Road, London.—Miss F. M. Long has been appointed night superintendent. She was trained at the Royal Hants County Hospital, Winchester, where she was afterwards sister, and she has since been night sister at Salop Infirmary, Shrewsbury.

Salisbury Hostel, Rhodesia, South Africa.—Miss Lillian E. Allen has been appointed maternity nurse. She was trained at the West Ham Infirmary, where she was afterward staff nurse and ward sister. Subsequently she was charge nurse at Long Reach Small-pox Hospital, Dartford, Kent; charge nurse at the Eastern Fever Hospital, Homerton, N.E.; and midwife at St. George's Workhouse, Buckingham Palace Road, S.W.

Tetbury Cottage Hospital.—Miss Alice Edith Keeble has been appointed matron. She was trained at the Royal Albert Edward Infirmary, Wigan, and she has filled various staff posts in that institution since the completion of her training. For the last three years she has been sister-in-charge of the operating theatre.

Wrexham Joint Fever Hospital.—Miss Margaret O'Neill has been appointed staff nurse. She was trained at Belfast Union Infirmary, where she has since been staff nurse. She has also been attached to a nursing institute at Cheltenham.

### The Nurses' Bookshelf.

A Manual for Nurses on Abdominal Surgery. By Harold Burrows, M.B., F.R.C.S. London. (The Scientific Press, Limited. Pp. 142. Illustrated. Price 2s. 6d. net.)

The education of nurses proceeds apace. It was formerly sufficient if they could make a bed, light a fire, and give the doctor a plain answer as to whether the patient had slept and eaten. Nowadays a nurse is as highly trained in her own department as is the attending medical man in his profession. She knows not only the names of the diseases, but often a great deal about their signs and symptoms. She can observe details, and give an absolutely reliable account of her observations. This book on abdominal surgery helps her still further. It teaches her the causes of the signs and symptoms that she is called upon to note. It is written clearly and concisely by one who is evidently an excellent surgeon endowed with the gift of teaching, for it is expressed in clear and simple language, whilst the information is both sound and practical.

Mr. Burrows deals first with the symptoms common to most forms of abdominal disease, and points out the features which distinguish dangerous conditions from those of minor importance. He then considers the subjects of peritonitis, hernia, obstruction of the bowels, affections of the liver and pelvic organs so far as they come within the purview of the general surgeon. There is a chapter on the methods of preparing a patient for abdominal section and another chapter on the after-troubles and complications of abdominal operations. The book ends with some suggestions on feeding. There is a sufficient index and some diagrams by way of rendering the text more easy to follow. The book is easy to read and contains much that is useful; it ought, therefore, to be of very great value to nurses as a guide to them in the execution of a difficult and responsible section of their work.

### A Book and its Story.

### A CANADIAN ROMANCE.*

Curtis Yorke's name on the cover of a novel is a guarantee that it is readable, true to life in incidents and characterisation, has bright dialogue, and a hero and heroine who, at least, are sufficiently natural to be interesting. The book before us has all these commendable qualities. The scene of it is laid in Canada, and the few characters that go to make it are admirably drawn. First, there is the girl heroine, Christian Cunninghame, a plucky little person who has been left an orphan and almost penniless, living in London in charge of an old servant named Milly, until an uncle sends for her to join him on his claim in Canada. He had prospected the claim for silver-lead, and found it. After four or five years' ownership the Carolay Claim became a fairly paying property. It was then he sent for her, and her maid to join him. Upon Christian's arrival he instructed her in the business of silver-lead mining. She mastered the details quickly, and at his death, which occurred three years later, in consequence of the shock caused by the discovery that the chief lode was dwindling and in danger of final disappearance, she set herself to fulfil his dying wish, that she should devote her life to the finding of the lost lode. This is her position when the book begins. She is living alone with her maid, directing the working of her property with an objectionable man as manager, employed by her merely because no one has offered who is better able to take charge of it at present. A small orphan boy known as "Gaddy," whose forlorn condition has appealed to her, is a member of the household also. He is a unique specimen of precocious childhood. His quaint sayings, always near the truth, and their unconscious humour are delightful. His first appearance is made on the occasion of the hero, Evan Warwick's passing through a valley on the property of Christian Cunninghame. Gaddy meets him. Warwick has bought the adjoining claim, and it has been discovered that the lost lode of silver-lead has reappeared in it; this Warwick at present knows nothing of, nor of the owner of the neighbouring property, Christian Cunninghame. Gaddy resents intruders and regards Warwick as one. Warwick, "keen-eyed, broad-shouldered, and generally good to look at," is riding slowly along the valley of Kootenay when he encounters the small and very consequential-looking little boy sauntering along with his hands in the pocket of his nankeen breeches, and an air of owning the universe. As Warwick comes within earshot, he is arrested by a shrill voice calling out, 'Hallo, there! Who are you? Put up your hands, my man. This is not a public road.'" Warwick's reply is to laugh, check his horse, and ask him to jump up on to the saddle in front of him. In this position they carry on a conversation, and Warwick gains information about Christian Cunninghame and her property. Gaddy is a valiant champion. On finding that Warwick is the new owner of the Barnethan Claim, he at first declines to have anything further to say to him. But having taken a fancy to Warwick, with a little diplomacy Warwick is able to find out about the Carolay Claim and its girl-owner. The boy gives his name as George Alander Duncan, usually called "Gaddy." He is an intimate friend of Miss Cunninghame's, and is astonished that Warwick has not heard of her. "'What! have you never heard of Miss Cunninghame?—Christian Cunninghame?' he exclaimed. . . . 'I don't believe you. Everyone knows about Miss Cunninghame!—Chris her name is. At least I always call her Chris. . . . She used to live in England, it's odd you did not know her in England.' 'England is not a nutshell, my son. Is she elderly, then, this person, this Miss Cunninghame?' 'Elderly!' echoed Gaddy. 'What, Chris? Well, I like that! Why she's just a girl, and no end pretty, I can tell you. At least *I* think so.' 'You're a first-rate champion, Gaddy; I shall look forward to seeing your friend some day. I did hear, now that I remember, that there was a mine a couple of miles from Barnethan. But I certainly did not understand it was owned by a woman.' 'Why, she runs the whole show. There is not much she cannot do, I can tell you. Ever since her mother died she has been all over the place. That's the kind of woman I mean to marry, when I'm a man.'" Very soon Christian herself appears. She has come out to look for Gaddy. The boy catches sight of her at once, and Warwick, following his gaze, looks towards a "lithe, girlish figure coming swiftly down a pathless rocky slope to his left." Shading her eyes with her hand, she called out in a voice of singular carrying power, "Is that you, Gaddy?" "I just shouldn't be surprised if it is," was the laconic reply. As the girl comes nearer Warwick is able to see that she is young, well made, and moderately pretty. Her face has a wonderfully alert look, which is in its way very attractive. She is wearing a workman-like costume; a dark blue skirt reaching barely to her neat ankles was surmounted by a crimson blouse clasped at the waist by a broad leather belt. Stout but trim boots and gaiters seemed to defy the roughness of the road by which she had come. A flat cap of faded red was pulled well over her forehead. Gaddy does not dismount on seeing her, and says, casually: "It's the Barnethan man, Chris. . . . But he's not a bit like what we thought he would be. He's a real good sort." Warwick dismounts and lifts his cap in silence. Christian receives his salute with hauteur, and addresses him with studied coldness. "You are the owner of the Barnethan claim?" "Yes," he answered, quietly, "I have bought the Barnethan claim. . . . We are fellow-countrymen, I hear," he continued, "so I hope we may be friends as well as neighbours. I am prepared to look on you and my new friend Gaddy as next-door neighbours," said Warwick, ignoring all possible unpleasantness. "I am afraid I have no time to make new friends," she returned, loftily. "I am a very busy woman, and as for Gaddy, he is a very naughty, disobedient little boy, and I daresay will do as he pleases." And with a stiff little bow she turned and went indoors, leaving Warwick not quite sure whether he was most angry or amused. Warwick walked away, a prey to conflicting emotion. 'Little spitfire,' he muttered. 'Confound her and her snuff-the-moon airs. Let it be war, then.' Christian, too, had determined that it should be war. However, it had been a decided shock to her that the much-discussed and long-expected enemy should have proved so provokingly personable, and so eminently desirable in every way." As a character-study Christian Cunninghame is unusually interesting and an admirable specimen of a type of woman dear to the author. Frank, brave, sensitive, and self-controlled. The love story of Evan Warwick and herself is a real romance, free from sentimentality and mock heroics, good to read and good to remember.

---

* "The Girl and the Man." By Curtis Yorke. (John Long. 6s.)

### Notes and Queries.

**REGULATIONS.**

**The Editor is always willing to answer in this column, without any fee, all reasonable questions, as soon as possible.**

**But the following rules must be carefully observed.**

1. **Every communication must be accompanied by the name and address of the writer.**
2. **The question must always bear upon nursing, directly or indirectly.**

**If an answer is required by letter a fee of half-a-crown must be enclosed with the note containing the inquiry.**

*Under Age.*

(194) Can I be received at any hospital infirmary or fever hospital at the age of 18?—*Clafton.*

No hospital, infirmary, or fever hospital will receive you so young. It is much wiser to wait until you are 23, and then join one of the large hospitals. Thus you will be eligible for good posts in the future. You can join a children's hospital at 21 for preliminary training if you wish.

*Homes for Gentlewomen.*

(195) Can you give me the address of the Nursing Home for Gentlewomen mentioned in your "Queries" column recently?—*Nottingham.*

We cannot identify the home you refer to, but you would do well to consult "Homes and Hospitals for the Benefit of Gentlewomen," published by the Army and Navy Co-operative Society, and obtainable from The Scientific Press, 28 and 29 Southampton Street, Strand, London, W.C., post free 1s. 1d.

*Nutriment per Rectum.*

(196) At the conclusion of a lecture given to nurses, a question was raised as to the quantity and method of preparing a simple nutrient, to be given per rectum, also the amount which should be given if a glycerine enema were ordered. The replies were so diverse and conflicting that I should feel grateful if the question might be answered in your columns.—*Inquirer.*

This is a matter about which nurses should always consult the medical man in charge of the case.

*Sterilised Dressings.*

(197) Can you tell me how to sterilise dressings without the proper apparatus.—*Sister.*

The simplest way is to boil them.

*Home in Italy.*

(198) Do you know of a home in or near Bologna, Italy, which would receive an Englishman between 60 and 70 years of age, who is suffering from creeping paralysis? His wife will not bring him to England.—*Etol.*

We do not know of any home such as you wish, but surely the wife will either see to this herself or prevent you from carrying out any project on her husband's behalf?

*Stewardess.*

(199) Has a nurse any chance of securing a post as stewardess on a passenger steamer?—*Berks.*

There are many more applications than posts for trained nurses on board ship. If you wish to undertake an ordinary stewardess's duties you might find a post, but the life has many disadvantages. Apply to any large steamship company.

*Woodwool.*

(200) Is there any place where I can buy woodwool cheap for a poor woman suffering from cancer?—*Caister.*

Possibly if you stated the case to a dispensary or hospital you might be allowed to get the woodwool cheaper, as these institutions buy in large quantities.

**Handbooks for Nurses.**

| | Post Free. |
|---|---|
| "A Handbook for Nurses." (Dr. J. K. Watson) ... | 5s. 4d. |
| "How to Become a Nurse." (Sir Henry Burdett) ... | 2s. 4d. |
| "Nurses' Pronouncing Dictionary of Medical Terms" | 2s. 0d. |
| "Art of Massage." (Creighton Hale) ... ... ... ... | 6s. 0d. |
| "Surgical Bandaging and Dressings." (Johnson Smith.) ... ... ... ... ... ... | 2s. 0d. |
| "Surgical Instruments and Appliances Used in Operations," illustrated. (Harold Burrows, F.R.C.S.) | 1s. 8d. |
| "Hints on Tropical Fevers." (Sister Pollard.) ... | 1s. 8d. |

Of all booksellers or of The Scientific Press, Limited, 28 & 29 Southampton Street, Strand, London, W.C.

### For Reading to the Sick.

OUR UNSEEN GUIDES.

Hand in hand with angels
  Through the world we go;
Brighter eyes are on us
  Than we blind ones know.
Tenderer voices cheer us
  Than we deaf will own;
Never walking heavenwards
  Can we walk alone.

*Lucy Larcom.*

He shall give His angels charge over thee to keep thee in all thy ways.—Ps. xci. 11.

Thus does the Lord provide us with heavenly guides who watch over us in infancy, and go with us "in all our ways," to keep us from danger; to protect us from evil; to guide us in the path of truth; to help us in bearing our burdens; to strengthen our weakness; to comfort us in sorrow; to aid us in our conflicts with evil; to give us new hopes, new joys, and new life. Step by step, with untiring assiduity and patience; to and fro, halting where we halt, waiting while we wait, turning as we turn, yet ever striving to withdraw us from evil by the whole strength of our affection, they take us by the hand tenderly, and lead us lovingly in all our ways from the cradle to the grave.

*Rev. Chauncey Giles.*

At death, when we die we have the dear angels for our escort on the way. Those who can grasp the whole world in their hands, can surely also keep our souls, that they journey safely home.—*Luther.*

As it is given us in the night of this world to behold the heavens studded with stars, great, glorious, and beautiful, in like manner has Scripture opened to our view a sight of the blessed angels. They appear as stars around us. But no unconcerned spectators in their silent watches Michael, "who is as (like unto) God?"; Gabriel, "the strength of God"; Raphael, "the healing of God" (so their names signify). They are ministering spirits sent by Him, shadows of His presence; He has revealed to us their deep concern for our welfare, their active ministrations about us day and night, and especially their peculiar regard for those who are of a meek spirit and despised of the world. What a dignity does this shed on our daily life!—*J. Williams.*

Here we have summed up the society in our future home. We shall then enjoy the society of the angels. We know about these holy beings, but we do not know themselves as yet. But how often does it happen to us in regard to our earthly friends, that those who are unknown to us in our early years even by name, become in our later years indissolubly bound up with our history and our joy? And thus the angels, whom on earth we have never seen, will, nevertheless, when the manhood of our being is reached, become our intimate friends, and dear companions for ever.

*Dr. Norman Macleod.*

For the great eye that sees us never sleeps;
It has its ministering angels wheresoe'er
Existence is—beneath us, and above,
Around us, and within us, He has there
His delegates.

